# Recurrent miscarriage in a woman with congenital factor V deficiency: a case report

**DOI:** 10.1186/s12884-022-05273-y

**Published:** 2022-12-08

**Authors:** Mohammad Shirzadi, Amir Hossein Radfar, Mehdi Dehghani

**Affiliations:** 1 Department of Internal Medicine, School of Medicine, Isfahan University of Medical Sciences, Isfahan, Iran; 2 School of Medicine, Isfahan University of Medical Sciences, Isfahan, Iran

**Keywords:** Factor V deficiency, Recurrent miscarriage, Miscarriage, Blood coagulation disorders, Pregnancy, Case report

## Abstract

**Background:**

Factor V deficiency is a rare bleeding disorder that can be either congenital or acquired. Factor V deficiency mostly present with mucosal bleeding. Coagulation factor V does not increase considerably during normal gestation. Since pregnancy can be threatened by blood clotting disorders, abnormal changes in coagulation factors level can pose challenges to pregnant women.

**Case presentation:**

We report a 40-year-old pregnant woman with prolonged gingival bleeding and epistaxis at 28 weeks of pregnancy. Her past medical history included two unexplained abortions. Physical examination was unremarkable, but the blood test showed elevated PT and PTT with a considerable decrease in factor V activity, while other factors were within normal range. Subsequently, the patient was diagnosed with congenital factor V deficiency. After treatment with fresh frozen plasma, she underwent vaginal delivery and a baby with factor V deficiency was born.

**Conclusions:**

This is the second report of recurrent miscarriage in congenital factor V deficiency patients. Clinicians should consider the possibility of factor V deficiency in women with a history of idiopathic miscarriage even in patients without any symptoms.

## Background

Factor V deficiency (FVD) is a rare blood coagulation disorder that can be classified into inherited or acquired [1]. The congenital form, with the inheritance pattern of autosomal recessive, affects around 1 per million population and is more prevalent in Muslim countries due to consanguineous marriages [2]. Its common clinical manifestations are mucocutaneous bleeding such as epistaxis, menorrhagia, and oral bleeding [3, 4]. According to factor V (FV) activity, this disease is divided into three types: mild (FV level ≥ 10%), moderate (FV level < 10%), and severe (FV level is undetectable) [1, 2].

As bleeding disorders can represent challenges to pregnancy and labor in women, certain abnormalities of coagulation cascade, e.g. deficiency of coagulation factors, should be considered in pregnant women [5–7]. Factors VII, VIII, X, XII, von Willebrand, and fibrinogen increase significantly during normal pregnancy. Factors II, V, and IX increase slightly or remain without changes but factor XI decreases [8, 9]. These changes lead to hypercoagulability and some imbalances in hemostasis of coagulation factors such as combined factor V + VIII deficiency may increase the risk of abortion [9, 10]. Due to the rarity of FVD, our literature review showed only one report of 6 cases with FVD and a single or recurrent miscarriage^1^[11]. Here, we describe a case of recurrent miscarriage in a patient with congenital factor V deficiency.

## Case presentation

A 40-year-old pregnant woman at 28th week of pregnancy was admitted to an obstetric emergency department in Isfahan, Iran, with chief complaints of epistaxis and gingival bleeding that were manifested about a week before admission.

She had no previous family history of bleeding disorders. But in her past medical history, it is highly notable that she had several hours of bleeding after eyebrow tattooing about 5 years before admission and two episodes of abortion in the first pregnancy trimester, 4 and 2 years before the current pregnancy. Moreover, her first pregnancy was successful. There were no abnormal data pertaining to her first labor.

Her medications were aspirin due to previous pregnancy losses, daily perinatal multivitamins, and iron supplements. She did not smoke or drink alcohol. Considering the patient’s condition, aspirin administration was discontinued and her symptoms reduced in intensity.

On admission, her vital signs were blood pressure 120/70, pulse 82, temperature 37 degrees C, respiratory rate 17. A detailed physical examination was performed but all findings were normal and no evidence of bleeding was found.

In her laboratory data, platelet count was in the normal range but prothrombin time (PT) and partial thromboplastin time (PTT) were both significantly elevated, so a mixing test was done. In the mixing test, both PT and PTT corrected instantly and after two hours of incubation at room temperature. Based on these results, the patient was suspected of having a deficiency of one or more coagulation factors. Thus, the activity level of coagulation factors were tested (Table 1). Laboratory results showed that the activity level of factor V was decreased significantly and the patient was diagnosed with factor V deficiency. In accordance with the mixing test result, the congenital form of FVD was approved.

**Table 1:** Patient’s laboratory values of coagulation factors

Coagulation factor	Lab value (%)	Normal range
Factor II	106	70–120
Factor V	4	70–120
Factor VII	129	40–145
Factor VIII	110	50–150
Factor IX	141	60–150
Factor X	87	45–155

The patient had a history of two unsuccessful pregnancies and some complications were possible due to significantly elevated PT and PTT. Thus, the patient was started on fresh frozen plasma (FFP) every three days -two units each time (15–20 ml/kg bodyweight)- from week 30 of pregnancy until the parturition and she responded well with increase in FV activity to the normal range. At 40 + 2 weeks gestation, the vaginal delivery was done successfully and the baby was born with 3820 gr weight and Apgar score of 9 and 10 in 1^st^ and 5^th^ minute respectively. The newborn was tested for bleeding disorders and diagnosed with congenital FVD. As the patient and her husband were cousins, the infant was a case of congenital FVD with parental consanguinity.

## Discussion and conclusions

Congenital FVD is a rare bleeding disorder (RBD) that may cause pregnancy complications. In our review of the literature, we found only a report of one or more miscarriages in FVD patients [11]. Our patient and those reported by Naderi et al. are different in some features. Contrary to our patient, prior cases had a history of parental consanguinity. In the other study, 3 of 6 patients had at least one successful delivery without medical intervention identical to the present case. In their patients, clinical features were epistaxis, gingival bleeding, menorrhagia, and deep soft tissue hematoma, but our patient only presented with epistaxis and gingival bleeding. In both cases, patients developed moderate FVD (based on FV activity). Notably, in two previous abortions of our patient, no symptom that may attribute to FVD was found which made it challenging to diagnose. It is debatable how some FVD patients have the history of recurrent miscarriage and also at least one successful pregnancy as seen in both reports. One possible hypothesis is the variable presentations of factor V deficiency (in low or undetectable FV levels); for example, many patients with severe FVD may bleed less than expected [12]. The mechanism of this finding has not been elucidated yet, but residual platelet factor V is suggested [13].

Some other disorders can also lead to bleeding during pregnancy including disseminated intravascular coagulation (DIC), thrombocytopenia, factor V leiden and liver abnormalities [14–17]. In order to check the existence of these diseases, D-dimer, fibrin degradation product (FDP), platelet count, aspartate aminotransferase (AST), and alanine aminotransferase (ALT) level were checked and they were within normal lab range (Table 2). Finally, test results confirmed congenital FVD in the patient.

**Table 2:** Patient’s laboratory data of coagulation, hematologic, metabolic and immunologic factors

Lab data	Lab value	Normal range
Platelets	157	145–450 × 10^3^/µL
Factor V Leiden (APC-R)	> 300	> 120 Seconds
D-dimer	170	0–200 µg/L
AST	18	0–31 IU/L
ALT	13	0–31 IU/L
Alkaline Phosphatase	169	64–306 IU/L
FDP	< 5	0–5 µg/ml
ANA	0.32	< 1.0%
Anti Cardiolipin IgG	5.3	< 12 GPL/ml
Anti Cardiolipin IgM	3.4	< 12 MPL/ml
Anti Phospholipid IgG	2	< 12 U/ml
Anti Phospholipid IgM	1.8	< 12 U/ml
Lupus Anticoagulant (DRVVT ratio)	0.9	< 1.2

Abbreviations: *AST* Aspartate Aminotransferase, *ALT* Alanine Aminotransferase, *FDP* Fibrin Degradation Products, *ANA* Anti Nuclear Antibody, *DRVVT* Dilute Russell Viper Venom Time

There are studies supporting the idea of an association between coagulation disorders and pregnancy loss [18–20]. Diagnosis of FVD and history of two miscarriages in our patient indicate that recurrent miscarriage may correlate with FVD. However, it was essential to investigate other possible causes of recurrent miscarriage in the patient. Several risk factors can contribute to recurrent miscarriage, including chromosomal and gene disorders, anatomical factors, endocrine abnormalities, immunological disorders, infectious diseases, etc. [20]. As a result of tests, infections, thyroid problems, diabetes, lupus, and antiphospholipid syndrome were all excluded in the patient (Table 2). Moreover, fetal aneuploidy is the most common cause of first trimester miscarriage [20]. Thus, cytogenetic testing of products of conception was performed in both previous pregnancy losses and revealed no findings suggesting aneuploidy or other chromosomal abnormalities. Therefore, our hypothesis about the relevance between congenital FVD and recurrent miscarriage was strengthened.

Moreover, choosing treatment strategy in pregnant women with FVD is important. To prevent bleeding complications, FFP, solvent-detergent FFP (SD-FFP), platelet transfusion, and recombinant factor VIIa should be considered as available choices [21, 22]. Some studies indicate that FFP administration before and during pregnancy is beneficial to reduce the risk of miscarriage [23, 24]. However, there are a few reports on patients who underwent caesarean delivery and successfully managed by platelet transfusion [25, 26].

In conclusion, this report and the previous one suggest that FVD can be a possible cause of recurrent miscarriage. Consequently, in patients that common causes of abortions are ruled out by assessment, obstetricians should regard FVD as a suspected diagnosis, even if it is asymptomatic. Early diagnosis is very important since some prescribed drugs for decreasing the risk of miscarriage (e.g. aspirin and heparin) may interfere with blood clotting action and worsen the underlying condition [27].

## Data Availability

All relevant data used or analyzed during this study are included in the manuscript.

## Abbreviations

FV: Factor V
FVD: Factor V deficiency
RBD: Rare bleeding disorder
PT: Prothrombin Time
PTT: Partial Thromboplastin Time
FDP: Fibrin degradation product
AST: Aspartate aminotransferase
ALT: Alanine aminotransferase
DRVVT: Dilute Russell Viper Venom Time
DIC: Disseminated intravascular coagulation
FFP: Fresh frozen plasma
SD-FFP: Solvent-detergent fresh frozen plasma

## Footnotes

1 Recurrent miscarriage is defined as having two or more miscarriages by ACOG (as considered in this report), while RCOG defines it as the loss of three or more consecutive pregnancies.

## References

[1] Tabibian S, Shiravand Y, Shams M, Safa M, Gholami MS, Heydari F (2019). A comprehensive overview of coagulation factor V and congenital factor V deficiency. Semin Thromb Hemost.

[2] Asselta R, Peyvandi F (2009). Factor V deficiency. Semin Thromb Hemost.

[3] Huang JN, Koerper MA (2008). Factor V deficiency: a concise review. Haemophilia.

[4] Dorgalaleh A, Alavi SER, Tabibian S, Soori S, Moradi E, Bamedi T (2017). Diagnosis, clinical manifestations and management of rare bleeding disorders in Iran. Hematology.

[5] Kadir R, Chi C, Bolton-Maggs P (2009). Pregnancy and rare bleeding disorders. Haemophilia.

[6] Bannow BS, Konkle BA (2018). Inherited bleeding disorders in the obstetric patient. Transfus Med Rev.

[7] Gernsheimer TB (2016). Congenital and acquired bleeding disorders in pregnancy. Hematol Am Soc Hematol Educ Program.

[8] Chi C, Kadir RA (2012). Inherited bleeding disorders in pregnancy. Best Pract Res Clin Obstet Gynaecol.

[9] Pike GN, Bolton-Maggs PHB (2011). Factor deficiencies in pregnancy. Hematol Oncol Clin North Am.

[10] Fogarty H, Doyle MM, Campbell R, Keenan C, White B, Ryan K (2019). Management of combined factor V and factor VIII deficiency in pregnancy. J Obstet Gynaecol.

[11] Naderi M, Tabibian S, Shamsizadeh M, Dorgalaleh A (2016). Miscarriage and recurrent miscarriage in patients with congenital factor V deficiency: a report of six cases in Iran. Int J Hematol.

[12] Duckers C, Simioni P, Spiezia L, Radu C, Gavasso S, Rosing J (2008). Low plasma levels of tissue factor pathway inhibitor in patients with congenital factor V deficiency. Blood.

[13] Duckers C, Simioni P, Spiezia L, Radu C, Dabrilli P, Gavasso S (2010). Residual platelet factor V ensures thrombin generation in patients with severe congenital factor V deficiency and mild bleeding symptoms. Blood.

[14] Erez O, Mastrolia SA, Thachil J (2015). Disseminated intravascular coagulation in pregnancy: insights in pathophysiology, diagnosis and management. Am J Obstet Gynecol.

[15] Greenberg EML, Kaled ESS (2013). Thrombocytopenia. Crit Care Nurs Clin North Am..

[16] Hamedi B, Feulefack J, Khan A, Sergi C (2020). Association between factor V Leiden mutation and recurrent pregnancy loss in the middle east countries: a Newcastle-Ottawa meta-analysis. Arch Gynecol Obstet.

[17] Sergi C, Al Jishi T, Walker M (2015). Factor V Leiden mutation in women with early recurrent pregnancy loss: a meta-analysis and systematic review of the causal association. Arch Gynecol Obstet.

[18] Rey E, Kahn SR, David M, Shrier I (2003). Thrombophilic disorders and fetal loss: a meta-analysis. Lancet.

[19] Barut MU, Bozkurt M, Kahraman M, Yıldırım E, Imirzalioğlu N, Kubar A (2018). Thrombophilia and recurrent pregnancy loss: the enigma continues. Med Sci Monit.

[20] Garrido-Gimenez C, Alijotas-Reig J (2015). Recurrent miscarriage: causes, evaluation and management. Postgrad Med J.

[21] Management of Inherited Bleeding Disorders in Pregnancy (2017). Green-top guideline no. 71 (joint with UKHCDO). BJOG Int J Obstet Gynaecol..

[22] Dorgalaleh A, Tabibian S, Hosseini MS, Shams M (2020). Pharmacological management of rare coagulation factor deficiencies besides hemophilia. Expert Rev Hematol.

[23] Younesi MR, Aligoudarzi SL (2013). Successful delivery in patients with severe congenital factor V deficiency: a study of five homozygous patients. Haemophilia.

[24] Shapiro A (2020). The use of prophylaxis in the treatment of rare bleeding disorders. Thromb Res.

[25] Derwall M, Grottke O (2021). Coagulation management for a caesarean delivery in a mother with severe homozygous factor V deficiency. J Clin Anesth.

[26] Drzymalski DM, Elsayes AH, Ward KR, House M, Manica VS (2019). Platelet transfusion as treatment for factor V deficiency in the parturient: a case report. Transfusion.

[27] de Jong PG, Kaandorp S, Di Nisio M, Goddijn M, Middeldorp S (2014). Aspirin and/or heparin for women with unexplained recurrent miscarriage with or without inherited thrombophilia. Cochrane Database Syst Rev.

